# Effect of γ-PGA and γ-PGA SAP on soil microenvironment and the yield of winter wheat

**DOI:** 10.1371/journal.pone.0288299

**Published:** 2023-07-14

**Authors:** Jianzhong Guo, Jingjing Zhang, Kangping Zhang, Sen Li, Yongkang Zhang

**Affiliations:** 1 Department of Art and Design, Taiyuan University, Taiyuan, Shanxi Province, China; 2 Taiyuan River and Lake Management Center, Taiyuan, Shanxi Province, China; 3 College of Water Resources Science and Engineering, Taiyuan University of Technology, Taiyuan, Shanxi Province, China; Central Research Institute for Dryland Agriculture, INDIA

## Abstract

Agricultural poly-γ-glutamic acid (γ-PGA) and γ-PGA super absorbent polymer (SAP) are two forms of γ-PGA applied in agriculture. Different quantities of γ-PGA and γ-PGA SAP (40 kg/hm^2^, 80 kg/hm^2^, 120 kg/hm^2^ and 160 kg/hm^2^) were applied to the soil in order to investigate their effects on the microenvironment of soil root zone and the yield of winter wheat. The soil water content increased with increasing amounts of γ-PGA SAP. The content of nitrate nitrogen and ammonium nitrogen increased with the increasing amounts of γ-PGA, while γ-PGA SAP significantly increased the content of ammonium nitrogen. The number of soil microorganisms and soil enzyme activities in the root zone increased with the addition of γ-PGA and γ-PGA SAP. The yield of winter wheat increased with the addition of γ-PGA or γ-PGA SAP, but the increasing rate decreased when the amount of γ-PGA and γ-PGA SAP exceeded 80 kg/hm^2^, with increases of 5.95% and 6.77% compared to the control group, respectively. The addition of γ-PGA significantly increased the protein content of wheat grains, and the WUE increased with increasing amounts of γ-PGA and γ-PGA SAP.

## Introduction

Poly-γ-glutamic acid (γ-PGA) is a type of polypeptide polymer, which a traditional Japanese food found in Natto [1]. It is different from protein formed by α-amino and γ-carboxyl groups, as it is composed of D-glutamic acid and L-glutamic acid monomers [2]. The γ-PGA is one of the main components of many microbial capsules, which gives it excellent biocompatibility and environmental microbial degradability [3]. The main chain of γ-PGA contains a large number of amide bonds, so it can be degraded into short peptide γ-PGA and glutamic acid monomers by microorganisms and enzymes that are harmless to the environment [4]. Many scientists worldwide are focusing their attention on its synthesis and application, as it has significant potential in biomedicine, environmental protection, food, and cosmetics. Currently, γ-PGA can be produced on a large scale by microbial fermentation.

There are two main applications of γ-PGA in agriculture. On the one hand, it can be used as a fertilizer synergist in agricultural after a certain crude extraction or a stock solution of γ-PGA produced by microorganisms. Preliminary studies have shown that adding γ-PGA to the soil could increase crop yield, improve crop quality, and improve soil microenvironment [5]. The secretion γ-PGA produced by two kinds of Bacillus subtilis was applied to potted maize in arid areas, and it was found that γ-PGA could improve the emergence rate of maize under drought conditions, improve the survival rate of maize seedlings and increase maize biomass, promote the growth of plant roots and leaves [6]. Applying agricultural γ-PGA to cotton planting under film drip irrigation in arid areas of Xinjiang could promote cotton growth, increase cotton yield, improve cotton quality, and improve soil water use efficiency. The best benefits were achieved at an application rate of 80 kg/ha [7]. The application of γ-PGA in the soil can significantly improve the growth of spinach, increase the fresh weight and dry matter yield of spinach, and improve the utilization efficiency of nitrogen fertilizer. The economic benefit is the best when the γ-PGA application amount is 0.10% [8]. The combined application of γ-PGA and nitrogen fertilizer can increase the soluble sugar content, free amino acid and protein content of rapeseed [9]. The application of γ-PGA biological water agent in soil can increase crop yield while reducing the amount of chemical fertilizer, and has a significant effect on the increase of soil microorganisms [10].

On the other hand, the super absorbent polymer (SAP) synthesized by the reaction of γ-PGA with a cross-linking agent or itself can be applied to agricultural water-saving irrigation as a soil water-retaining agent. The super absorbent polymer (SAP) is a kind of polymer material that can absorb hundreds or even thousands of times of water. The results have shown that γ-PGA SAP can inherit the biodegradability of γ-PGA. The γ-PGA SAP can be degraded by microorganisms in the environment after the final completion of water retention. It can eventually be decomposed into soil nitrogen fertilizer or can act as a fertilizer synergist because the monomer of γ-PGA SAP is glutamic acid. It can be transformed into fertilizer that can be used by crops through a series of fertilizer transformation. The degradability of the super absorbent polymer based on poly-glutamic acid is better than the commonly used poly-acrylate super absorbent polymer by C-O bond cross-linking polymerization. However, the super absorbent polymer synthesized based on poly-acrylate on the current market is not degradable or only partially degradable in the natural environment, which can easily to cause pollution in the environment.

Due to its recent development in agriculture, γ-PGA is a new type of microbial material. However, there are few studies on the agricultural application of SAP synthesized based on γ-PGA. Poly-acrylate SAP, on the other hand, has been more extensively studied in its application to agriculture. Seed dressing with SAP can increase seed emergence rates and ultimately boost crop yields between 50% to 80% of the field water holding capacity [11]. When 60 kg/hm^2^ SAPs were applied in the field, the yield of wheat increased 14.7% compared to the control group [12]. The application of SAP in soil could promote the development of maize roots, increases the number of roots in the maize root system, and significantly affects the yield components of maize, improving overall maize yields [13]. The boll number, boll weight and lint yield per unit area increased by over 10% when 30–45 kg/hm^2^ of SAP was used, but the fiber quality was not affected by the SAP [14]. In addition, the results showed that the application of 15 kg/hm^2^ SAP could increase the yield of potato by 16%, as well as improve the efficiency of phosphorus and nitrogen fertilizer utilization by 27.06% and 18.72%, respectively [15].

Both γ-PGA and SAP can improve crop yield to a certain extent. It should be noted that the molecular structure of γ-PGA is a single-chain, water-soluble polymer material, whereas the molecular structure of γ-PGA SAP is a three-dimensional network, water-insoluble polymer material. Thus, while both γ-PGA and γ-PGA SAP are derivatives of γ-PGA, the effects of γ-PGA and γ-PGA SAP on crops may not be consistent. This paper compares the effect of different levels of γ-PGA and γ-PGA SAP on the soil root zone microenvironment (soil water content, soil nitrate and ammonium nitrogen contents, soil microbial abundance and soil enzyme activities), the yield composition (grain number, grain weight and yield) and grain quality (protein, starch and reducing sugar) of winter wheat (*Triticum aestivum*) were compared when different levels of γ-PGA and γ-PGA SAP were applied to the soil.

## Materials and methods

### Experiment design and measurement index

**Fig 1:**
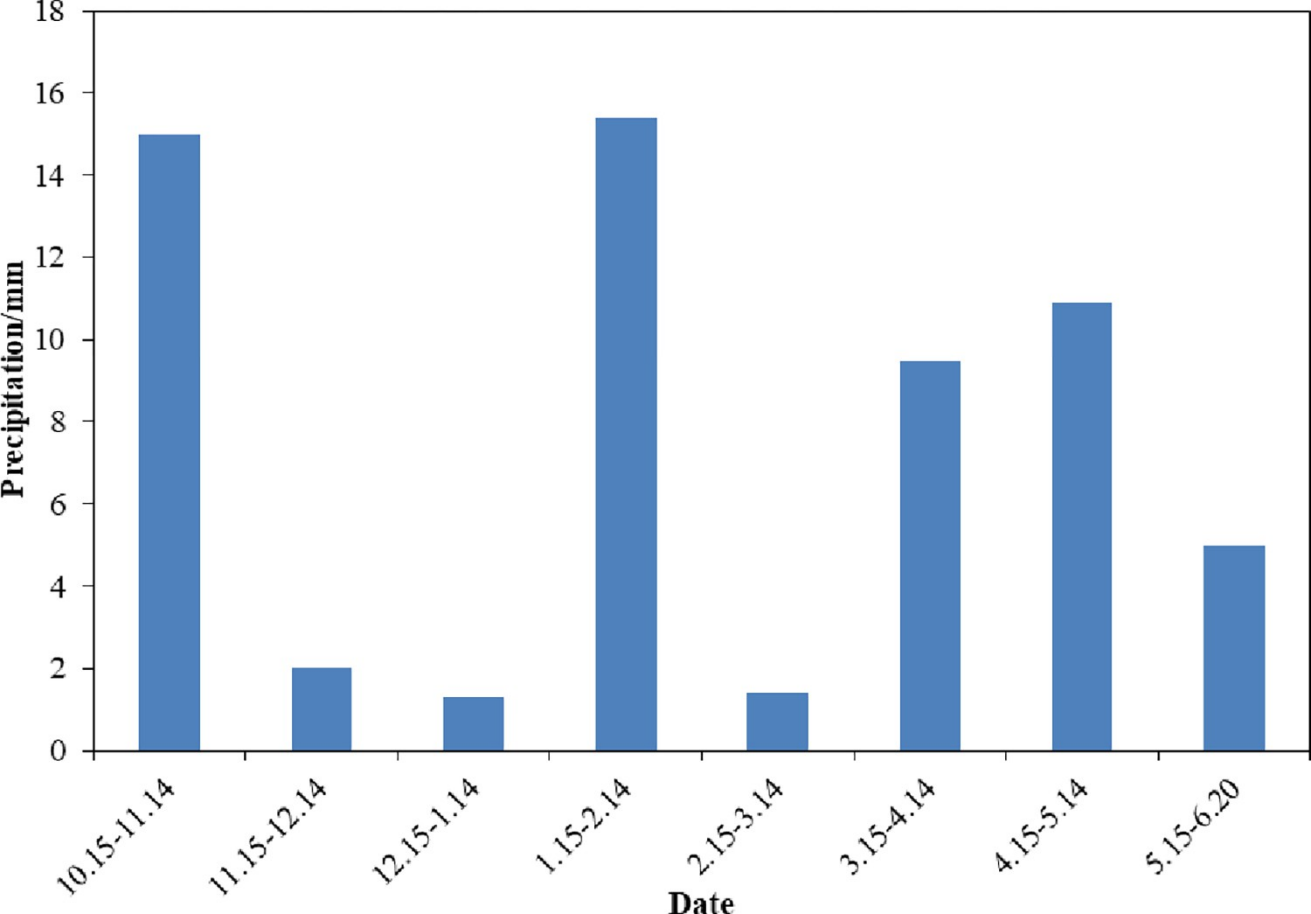
Precipitation amount.

The experiment was carried out at the Xiwenzhuang Township Irrigation Experiment Station, Taiyuan City, Shanxi Province from October 15, 2021 to June 20, 2022. The precipitation during this period is shown in Fig 1. The experiment area belongs to warm temperate continental climate, with an average annual rainfall of 430 mm, an average annual evaporation of 1812 mm, an annual average temperature of 9.5°C, a frost-free period of 170 days, and an annual average sunshine hours of 2676 h. The soil type is clay loam. The basic values of soil nutrients are shown in Table 1.

**Table 1:** Soil basic nutrients.

Indicators	Total N (g/kg)	Total P (g/kg)	Organic matter (g/kg)
Value	0.54	0.73	29.93

### Experiment design

The area of the experiment is 2 m×3 m. There was no need for irrigation before sowing as the soil water content was suitable for wheat growth. It only needed to plough the soil and apply base fertilizer. The amount of irrigation and fertilization during the experiment was carried out according to the local irrigation and fertilization system. Wheat was seeded on October 15 using row seeding (furrow seeding) with a row spacing of 0.2 m and each row being 3 m. The sowing depth was 3 cm, and the sowing rate was 225 kg/hm^2^. The wheat variety was Jinmai 1001. The wheat experiment was set up 9 treatments (1 control group, 4 levels of γ-PGA and 4 levels of γ-PGA SAP), each repeated three times. The experiment treatments are shown in Table 2.

**Table 2:** The design of experiment.

Serial number	Treatment	Additive agent	Addition amount
1	CK	no	0
2	P40	γ-PGA	40 kg/hm^2^
3	P80	γ-PGA	80 kg/hm^2^
4	P120	γ-PGA	120 kg/hm^2^
5	P160	γ-PGA	160 kg/hm^2^
6	PS40	γ-PGA SAP	40 kg/hm^2^
7	PS80	γ-PGA SAP	80 kg/hm^2^
8	PS120	γ-PGA SAP	120 kg/hm^2^
9	PS160	γ-PGA SAP	160 kg/hm^2^

The type of γ-PGA used in this experiment is powder form, which is produced by Shandong Freda Biotechnology Co., LTD. The type of SAP used is the γ-PGA SAP prepared by our laboratory, and the preparation method of SAP is shown in the paper [16, 17]. Both of agricultural γ-PGA and γ-PGA SAP are derivatives of γ-PGA which was used in agriculture field, due to the different properties of γ-PGA and γ-PGA SAP for agriculture, appropriate application methods are adopted to apply them to soil respectively. Agricultural γ-PGA was mixed well by rotary tillage, while γ-PGA SAP was mixed with seeds and fine sand (1:3 γ-PGA SAP: fine sand) for strip sowing. The amount of irrigation and fertilization in this experiment is based on the local conventional management system. The amount of irrigation is 270 mm [18]. The irrigation was carried out on April 5, April 25 and May 15, and the each amount of irrigation was 90 mm. In the experiment, 750 kg/hm^2^ compound fertilizer (N, P, K≥15%) was directly applied as the base fertilizer, and 225 kg/hm^2^ urea was applied during the jointing stage, which was equivalent to the recommended amount of local field.

### Collection of soil samples and determination of indexes

The standard growth period of wheat was observed when more than 50% of wheat reached the standards of seedling stage, regeneration stage, heading stage, filling stage and maturity stage (Table 3) [19].

**Table 3:** Division of growth stages of winter wheat in this experiment.

Stage	Seeding-tillering	Tillering- overwintering	Overwintering-regreening	Regreening-jointing	Jointing-heading	Heading-filling	Filling-maturity
Date	Oct 15 –Nov 25	Nov 26—Dec 4	Dec 5—Mar 21	Mar 22—Apr 21	Apr 22- May 10	May 11—May 28	May 29-Jun 29

In this experiment, the soil samples were collected during critical growth periods of wheat on November 10, February 20, April 1, April 30, May 20 and June 25. The soil samples were divided into two parts to measure soil water content and soil available nutrients, respectively. The soil samples were collected from different layers: 0–10 cm, 10–20 cm, 20–30 cm, 30–40 cm, 40–60 cm, 60–80 cm, and 80–100 cm. Average soil water content and average nutrient value of each layer were collected as treatment data for this growth period. Additionally, for soil root samples of wheat, the number of soil microorganisms and the activity of soil enzymes were determined from the 3–8 cm layer. The soil samples were stored in a refrigerator at 4°C and measured in a timely manner.

The soil water content was determined by the drying method. Soil samples were dried in an oven at 105°C for 8 hours, and the mass of the samples was measured on an aluminum box. Soil nitrate nitrogen was measured by UV spectrophotometer at dual wavelengths of 220 nm and 275 nm, while soil ammonium nitrogen was measured by indophenol blue colorimetric method by UV spectrophotometer at 625 nm. The number of soil bacteria, fungi and actinomycetes was determined by dilution plate counting method. The bacteria were cultured by beef extract peptone medium, the actinomycetes were cultured by modified Gaoshi No.1 medium, and the fungi were cultured by Martin medium. Each soil sample was repeated three times.

Soil enzyme activity was determined as follows: soil urease activity was measured by indophenol blue colorimetric method, soil alkaline phosphatase activity was measured by diphenyl phosphate colorimetry, soil sucrase activity was measured by 3, 5-dinitrosalicylic acid method.

### Determination of wheat yield and grain quality

The number of productive wheat ear was counted at harvest. Each treatment selected evenly growing 20 spikes of wheat, counting the number of grains per spike. The yield of each treatment was measured by threshing after harvest, and the 1000-grain weight of each treatment was measured.

The protein content of wheat flour was determined by Coomassie brilliant blue method, the starch content of wheat flour was determined by anthrone colorimetric method, and the reducing sugar content of wheat flour was determined by dinitrosalicylic acid method.

## Results and analysis

### Effects of γ-PGA and γ-PGA SAP on soil water content

**Fig 2:**
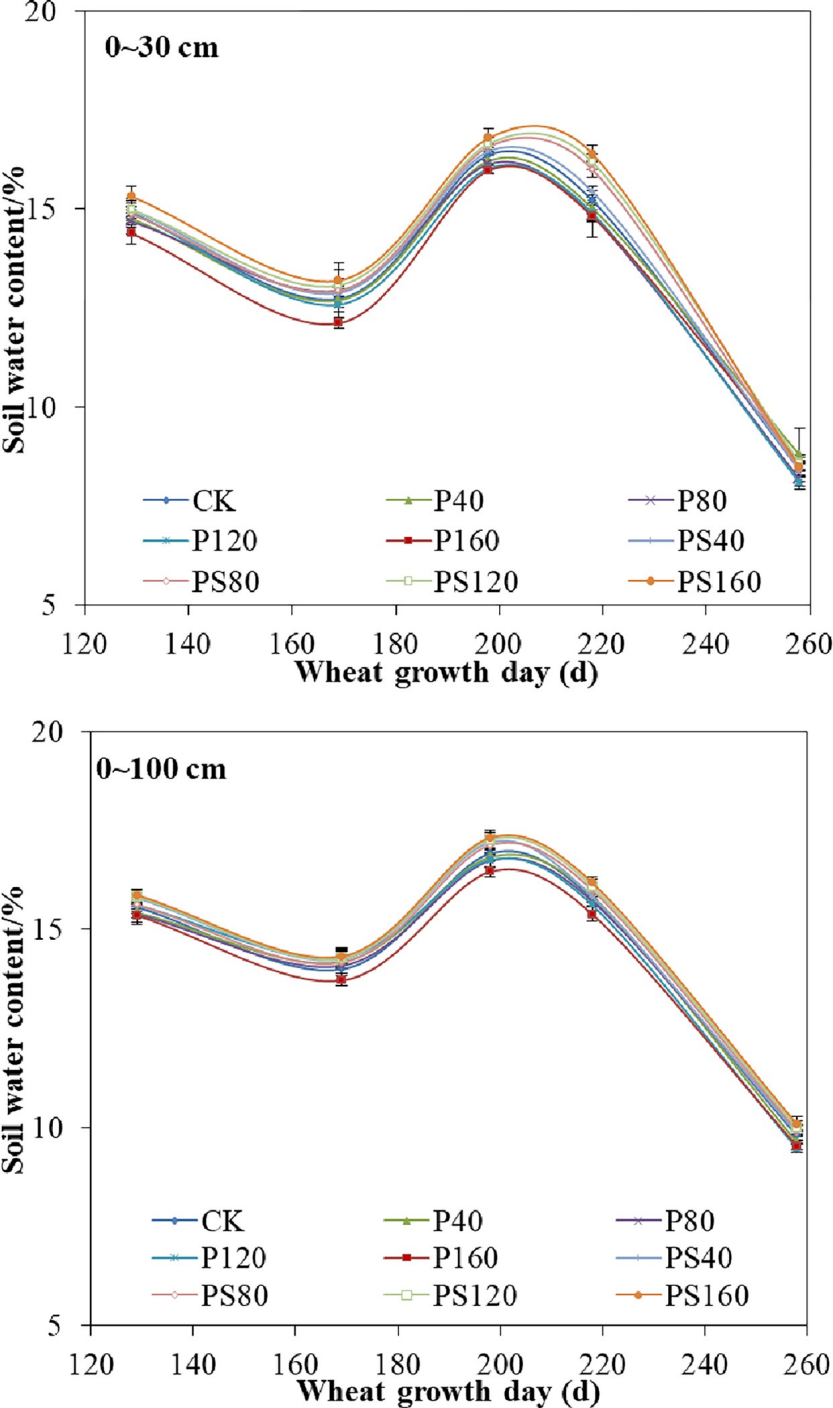
Effects of γ-PGA and γ-PGA SAP for soil water content. (Notes: The soil water content in the figure is the soil mass water content).

The response of different contents of γ-PGA and γ-PGA SAP added to the soil towards soil water content at different growth stages of winter wheat was shown in Fig 2. With the advancement of the growth period of winter wheat, the soil water content decreased before jointing stage. The soil water content increased from the jointing stage to the heading stage, and then the soil water content decreased after heading stage, mainly due to the irrigation period. The average soil water content decreased by -2.98%, -3.47%, -3.04% and -4.57% respectively with the increase of γ-PGA addition in the 0~30 cm depth of soil compared with CK. The average soil water content increased by 0.80%, 1.75%, 2.76% and 3.87% compared with CK with the increase of γ-PGA SAP content, respectively. The average soil water content decreased by -0.22%, -1.27%, -1.88% and -2.59% respectively with the increase of γ-PGA application in the 0~100 cm depth of soil compared with CK. The average soil water content increased by -1.91%, -1.75%, -0.88% and -0.45% compared with CK with the increase of γ-PGA SAP content, respectively. From the changes of soil water content in 0-30cm and 0-100cm layers, it can be seen that the soil water content in the surface layer varies greatly compared with the soil water content in the whole layer. This is because the soil water content in the surface layer is easily affected by additives in the soil layer and atmospheric environment. In this experiment, the increased soil water content treated with γ-PGA SAP compared with CK was lower than that of the field water capacity of the soil treated with γ-PGA SAP compared with field water capacity of the soil [20]. This is because that the field water capacity of the γ-PGA SAP was affected by the water absorption of wheat roots and buried in the soil layer of 3–8 cm, and the fertilizer applied in the soil will also have a certain impact on the water absorption of the γ-PGA SAP [21]. The average water content in the growth period decreased slightly when γ-PGA was applied to the soil [7]. While average soil water content in growth period increased with the increase of γ-PGA SAP content.

### Effect of γ-PGA and γ-PGA SAP on soil nitrate nitrogen content

**Fig 3:**
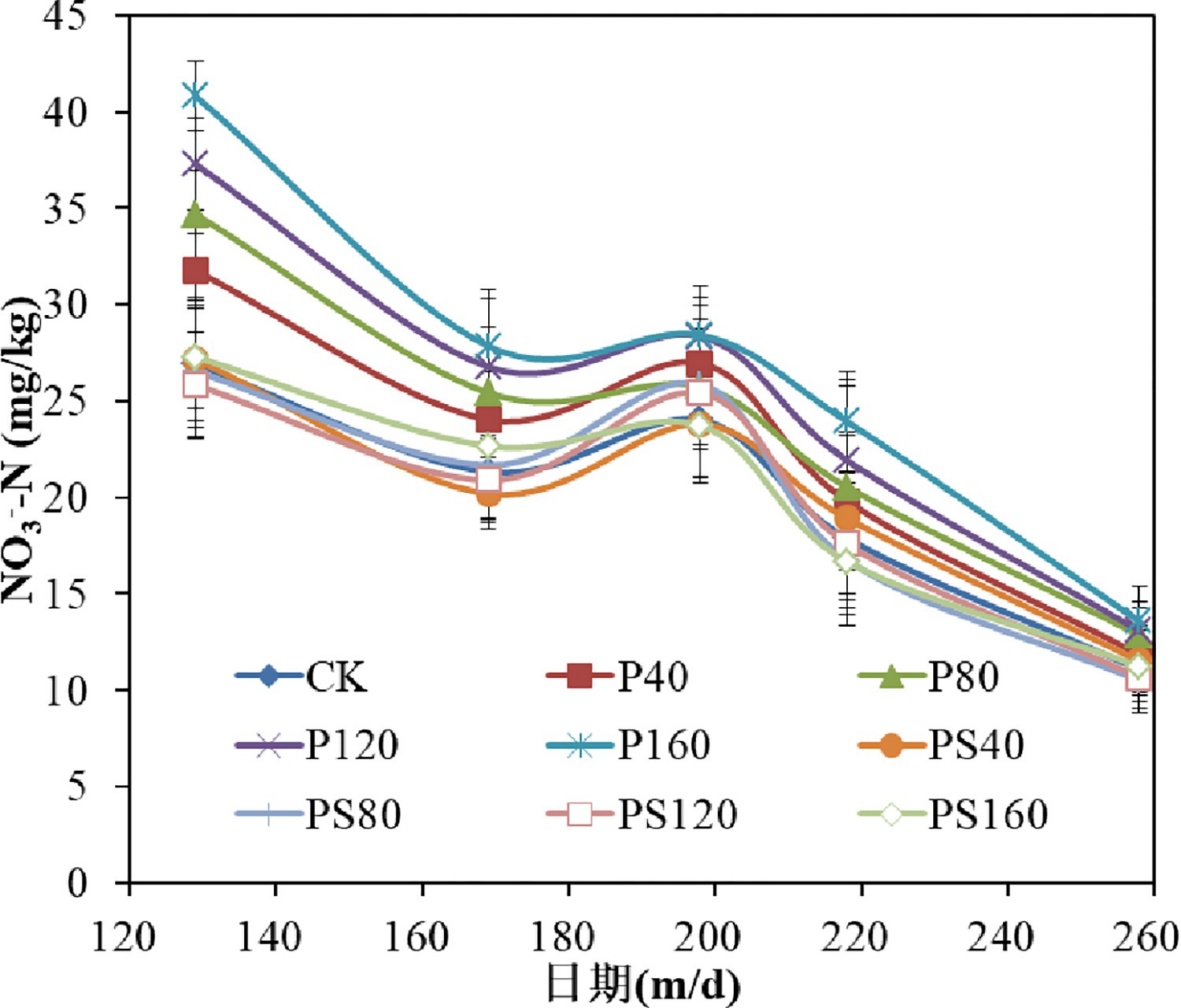
Effects of γ-PGA and γ-PGA SAP for soil nitrate nitrogen content.

The Fig 3 shows the responses of different amounts of γ-PGA and γ-PGA SAP to soil nitrate nitrogen at different growth stages of winter wheat. As can be seen from the figure, soil nitrate nitrogen showed a trend of initially decreasing and then rising in different treatments, which was mainly related to the fertilization cycle with the change of growth period. With the advance of growth period, the difference in soil nitrate nitrogen content among different treatments gradually decreased. This was due to the fact that the nitrate nitrogen in the soil was absorbed by the wheat roots after the fertilization stopped in the middle and late stages of wheat growth. The average soil nitrate nitrogen content in the growth period of each treatment increased by 11.26%, 13.83%, 21.33% and 26.25% with the increasing of γ-PGA content compared with CK, respectively, while the soil nitrate nitrogen content increased by -0.19%, 0.71%, 0.39% and -0.04% compared with CK with the increasing of γ-PGA SAP content compared with CK, respectively. The content of soil nitrate also significantly increased with the increase in γ-PGA content in the soil, but no significant difference was observed between the soil nitrate content and the control group.

### Effect of γ-PGA and γ-PGA SAP on soil ammonium nitrogen content

**Fig 4:**
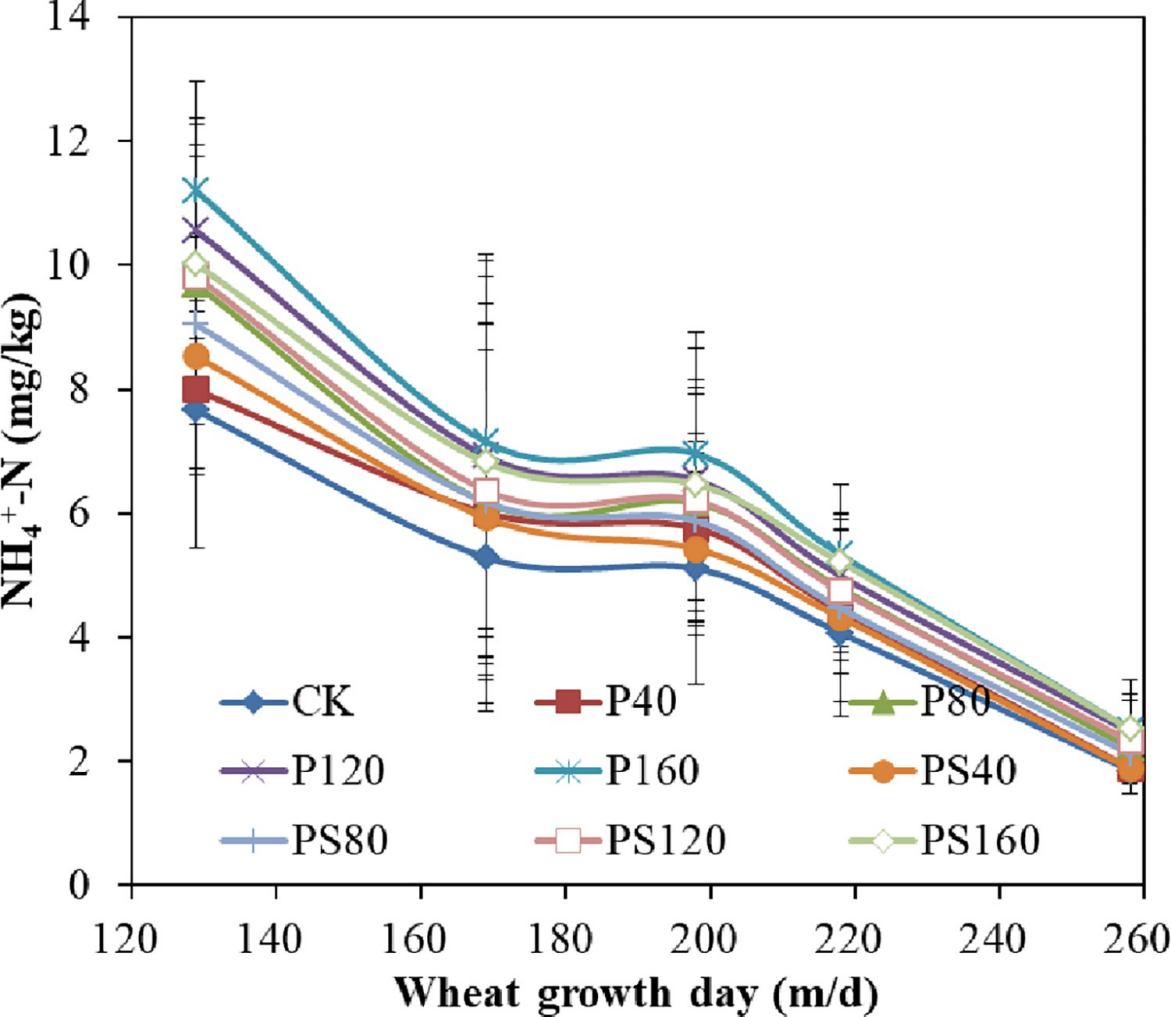
Effects of γ-PGA and γ-PGA SAP for soil ammonium nitrogen content.

The response of different amounts of γ-PGA and γ-PGA SAP to soil ammonium nitrogen during different growth stages of winter wheat was shown in Fig 4. The change in soil ammonium nitrogen content during the entire growth period was similar to that of soil nitrate nitrogen, as seen in Fig 3. However, the soil ammonium nitrogen content was lower than that of nitrate nitrogen in the soil. The content of soil ammonium nitrogen in each treatment increased initially and then decreased. This trend correlated with the fertilization cycle and amount, and was consistent with the change in soil nitrate nitrogen. As the wheat growth period progressed, the difference in soil ammonium nitrogen content between treatments decreased gradually. This decrease was due to the absorption of soil ammonium nitrogen by wheat roots and the volatilization of soil ammonium nitrogen after stopping fertilization in the middle and late stages of wheat growth [22]. The soil ammonium nitrogen content in each treatment increased by 8.67%, 21.02%, 31.23%, and 38.20% with the increase of γ-PGA content compared to CK. Similarly, the soil ammonium nitrogen content in each treatment increased by 8.80%, 15.47%, 22.89%, and 29.40% with the increase of γ-PGA SAP content compared to CK.

### Effect of γ-PGA and γ-PGA SAP on soil microorganism

The Table 4 showing the changes of different amounts of γ-PGA and γ-PGA SAP on soil microbial numbers (bacteria, actinomycetes and fungi) at maturity stage of winter wheat.

**Table 4:** The number and community index of microorganisms in root zone of γ-PGA and γ-PGA SAP.

Treatment	Bacteria (×10^9^ cfu)	Actinomycetes (×10^7^ cfu)	Fungi (×10^5^ cfu)	Shannon index	Simpson index
CK	0.45±0.24d	0.14±0.08c	0.41±0.43e	0.0219	0.0064
P40	1.13±0.48cd	0.93±0.62bc	0.95±0.42e	0.0482	0.0164
P80	1.86±0.57bc	1.56±0.65bc	2.24±0.64de	0.0493	0.0167
P120	2.72±0.88b	2.11±0.83b	3.58±0.74cd	0.0464	0.0155
P160	3.59±0.74a	4.72±2.44a	4.49±0.58bc	0.0705	0.0259
PS40	0.67±0.30d	0.31±0.21bc	1.43±0.72e	0.0314	0.0096
PS80	0.91±0.12cd	0.74±0.28bc	4.51±1.11bc	0.0511	0.0170
PS120	1.33±0.32cd	1.05±0.30bc	5.72±1.76ab	0.0495	0.0164
PS160	1.82±0.40bc	1.47±0.51bc	6.85±1.91a	0.0500	0.0166

The number of soil bacteria increased by 151.11% (P40), 313.33% (P80), 504.44% (P120) and 697.78% (P160), respectively, with the increase of γ-PGA content compared to CK. The number of soil bacteria increased by 48.89% (PS40), 102.22% (PS80), 195.56% (PS120) and 304.44% (PS160) with the increase of γ-PGA SAP content compared with CK, respectively. The number of bacteria applied with the same amount of γ-PGA in the soil at the maturity stage was 1.69 times (40 kg/hm^2^), 2.04 times (80 kg/hm^2^), 2.08 times (120 kg/hm^2^) and 1.97 times (160 kg/hm^2^) than the number of bacteria applied with the same amount of γ-PGA SAP in the soil. Therefore, applying the same amount of γ-PGA in the soil can increase the number of soil bacteria more than applying the same amount of γ-PGA SAP in the soil.

The number of soil actinomycetes increased by 564.29% (P40), 1014.29% (P80), 1407.14% (P120) and 3271.43% (P160) with the increase of γ-PGA content compared with CK, respectively. The number of soil actinomycetes increased by 121.43% (PS40), 428.57% (PS80), 650.03% (PS120) and 950.24% (PS160) with the increase of γ-PGA SAP content compared with CK, respectively. The number of actinomycetes applied with the same amount of γ-PGA in the soil at the maturity stage was 3.00 times (40 kg/hm^2^), 2.11 times (80 kg/hm^2^), 2.01 times (120 kg/hm^2^) and 3.21 times (160 kg/hm^2^) of the number of actinomycetes applied with the same amount of γ-PGA SAP in the soil. Therefore, the application of the same amount of γ-PGA in the soil can increase the number of soil actinomycetes more than the application of the same amount of γ-PGA SAP.

The number of soil fungus increased by 131.71% (P40), 446.34% (P80), 773.17% (P120) and 985.93% (P160) with the increase of γ-PGA content compared with CK, respectively. The number of soil fungus increased by 248.78% (PS40), 1000.00% (PS80), 1295.12% (PS120) and 1570.73% (PS160) with the increase of γ-PGA SAP content compared with CK, respectively. The number of fungus applied with the same amount of γ-PGA in the soil at maturity was 0.66 times (40 kg/hm^2^), 0.50 times (80 kg/hm^2^), 0.63 times (120 kg/hm^2^) and 0.66 times (160 kg/hm^2^) of the number of fungus applied with the same amount of γ-PGA SAP in the soil. Therefore, the application of the same amount of γ-PGA in the soil can increase the number of soil fungus less than the application of the same amount of γ-PGA SAP.

The number of microorganisms and community index in the soil root zone of winter wheat at maturity under different treatments was shown in Table 4. Biodiversity indexes are expressed through the Shannon index and Simpson index. In this experiment, the measured microbial species were bacteria, actinomycetes, and fungi. The Shannon index and Simpson index indicate the uniformity of the community. The higher value of the Shannon and Simpson index, the more uniform distributed of microbial species. As can be seen from the table, the Shannon index and Simpson index of γ-PGA and γ-PGA SAP treatment increased compared with the control group without the addition of γ-PGA or γ-PGA SAP. The Shannon index and Simpson index increased gradually with the increasing application amount of γ-PGA or γ-PGA SAP, indicating that the increasing of application amount of γ-PGA or γ-PGA SAP could promote the uniformity of microbial community. The Shannon index and Simpson index of the treatment with γ-PGA SAP were higher than those of the treatment with the same amount of γ-PGA which was due to the number of fungi in soil treated with γ-PGA SAP was higher than that treated with γ-PGA SAP (Table 4).

### Effects of γ-PGA and γ-PGA SAP on soil enzyme activities

The changes of γ-PGA and γ-PGA SAP on soil enzyme activities (urease, phosphatase and sucrase) at maturity of winter wheat was showed in Table 5.

**Table 5:** γ-PGA and γ-PGA SAP for soil enzyme activities.

Treatment	Urease activities	Phosphatase activities	Sucrase activities
CK	0.1247±0.0393d	1.4325±0.0464f	2.7483±1.2121e
P40	0.1756±0.0866cd	2.1668±0.7643def	8.2436±4.5840cde
P80	0.2455±0.0264bc	3.2739±0.3617bc	14.1132±4.2461bc
P120	0.3468±0.0907ab	3.8568±0.4395ab	18.9006±4.8500b
P160	0.4228±0.0205a	4.3587±0.3390a	26.9651±7.9627a
PS40	0.1524±0.0413cd	1.8364±0.3825ef	5.4191±0.5541de
PS80	0.2217±0.0409cd	2.4489±0.4269cde	7.5752±1.9384cde
PS120	0.3289±0.0612ab	2.9873±0.4625bcd	9.4216±1.9017cde
PS160	0.3974±0.0726a	3.5476±0.5391ab	11.6765±3.2539bcd

Table 5 displays the changes in urease activities observed in different treatments during maturity. With an increase in γ-PGA content, the urease activities under different treatments demonstrated an increment of 40.81% (P40), 96.85% (P80), 178.07% (P120) and 239.15% (P160) compared to CK. Similarly, the urease activities under different treatments increased by 4.11% (PS40), 15.38% (PS80), 25.51% (PS120) and 54.89% (PS160) when γ-PGA SAP content increased as compared to CK. At maturity stage, the urease activities with the same amount of γ-PGA in the soil were 1.15 times (40 kg/hm^2^), 1.11 times (80 kg/hm^2^), 1.05 times (120 kg/hm^2^) and 1.06 times (160 kg/hm^2^) higher than the urease activities attained with the same amount of γ-PGA SAP in the soil. Therefore, the application of the same amount of γ-PGA in the soil can increase the urease activities more than the application of the same amount of γ-PGA SAP.

The phosphatase activities in different treatments changed at maturity was showed in Table 5. The phosphatase activities under different treatments increased by 51.26% (P40), 128.54% (P80), 169.24% (P120) and 204.27% (P160) with the increase of γ-PGA content compared with CK, respectively. The phosphatase activities increased by 28.20% (PS40), 70.95% (PS80), 108.54% (PS120) and 147.65% (PS160) with the increase of γ-PGA SAP content compared with CK, respectively. The phosphatase activities applied with the same amount of γ-PGA in the soil at maturity was 1.18 times (40 kg/hm^2^), 1.34 times (80 kg/hm^2^), 1.29 times (120 kg/hm^2^) and 1.23 times (160 kg/hm^2^) of the phosphatase activities applied with the same amount of γ-PGA SAP in the soil. Therefore, the application of the same amount of γ-PGA in the soil can increase the phosphatase activities more than the application of the same amount of γ-PGA SAP.

The sucrase activities in different treatments changed at maturity was showed in Table 5. The sucrase activities under different treatments increased by 199.95% (P40), 413.52% (P80), 587.72% (P120) and 881.16% (P160) with the increase of γ-PGA content compared with CK, respectively. The sucrase activities increased by 97.18% (PS40), 175.63% (PS80), 242.82% (PS120) and 324.50% (PS160) with the increase of γ-PGA SAP content compared with CK, respectively. The sucrase activities applied with the same amount of γ-PGA in the soil at maturity was 1.52 times (40 kg/hm^2^), 1.86 times (80 kg/hm^2^), 2.01 times (120 kg/hm^2^) and 2.31 times (160 kg/hm^2^) of the sucrase activities applied with the same amount of γ-PGA SAP in the soil. Therefore, the application of the same amount of γ-PGA in the soil can increase the sucrase activities more than the application of the same amount of γ-PGA SAP.

### Effects of γ-PGA and γ-PGA SAP for wheat yield and quality

#### Effects of γ-PGA and γ-PGA SAP for wheat yield composition

The effect of different contents of γ-PGA and γ-PGA SAP on winter wheat yield indexes (number of panicles, grain number per ear, 1000-grain weight and yield) was showed in Table 6.

**Table 6:** Effects of γ-PGA and γ-PGA SAP for yield indexs and grain quality of winter wheat.

Treatment	Number of panicles (×10^4^)	Grains per ear	1000-grains (g)	Yield (kg/hm^2^)	Protein	Starch	Reducing sugar
CK	432.35±13.9b	35.31±0.62a	36.81±0.56a	5873.08±132.96b	9.44±0.67c	55.34±1.77a	1.13±0.22a
P40	483.77±20.2ab	34.83±0.52a	36.84±0.47a	6182.73±384.05 ab	9.72±0.51bc	56.12±2.11a	1.15±0.24a
P80	500.41±24.1ab	34.46±0.26a	37.21±0.62a	6327.35±298.69ab	10.04±0.44abc	54.25±1.04a	1.24±0.14a
P120	515.28±40.9a	35.58±0.75a	36.64±0.28a	6422.74±483.95a	10.43±0.26ab	58.18±3.37a	1.29±0.15a
P160	516.74±29.4a	35.92±1.20a	36.91±0.29a	6498.31±309.06a	10.79±0.33a	57.23±1.78a	1.31±0.26a
PS40	456.17±62.8ab	34.45±0.56a	36.85±0.41a	6008.38±356.05 ab	9.21±0.55c	57.37±1.81a	1.11±0.16a
PS80	471.62±54.4ab	34.88±0.52a	36.67±0.25a	6177.61±481.37 ab	9.54±0.58bc	55.92±2.20a	1.22±0.17a
PS120	494.24±49.1ab	35.28±0.46a	37.14±0.42a	6246.78±332.92 ab	9.63±0.23bc	56.61±2.23a	1.27±0.13a
PS160	501.16±28.5ab	35.84±1.59a	36.47±0.35a	6305.49±263.81 ab	9.92±0.37abc	56.93±1.88a	1.18±0.12a

The spike number refers to the total spike number per unit area of wheat, which can reflect its tillering and growth situation, and is one of the important indicators to gauge wheat yield. The number of panicles of winter wheat increased by 11.89% (P40), 15.74% (P80), 19.18% (P120) and 19.52% (P160) compared with CK with the increasing of γ-PGA application in the soil, respectively. The number of panicles of winter wheat increased by 5.51% (PS40), 9.08% (PS80), 14.31% (PS120) and 15.91% (PS160) compared to CK with the increasing of γ-PGA application in the soil, respectively.

The numbers of grain per ear and 1000-grain weight are directly related to the yield of winter wheat, and they are also one of the main indexes of winter wheat yield. The number of grains per spike showed an increasing trend with the increasing of γ-PGA or γ-PGA SAP content in the soil. The number of grains per ear with the same amount of γ-PGA in the soil was higher than that with γ-PGA SAP in the soil. The 1000-grain weight of wheat was not significantly affected by the application of γ-PGA or γ-PGA SAP in the soil.

The yield of wheat increased with the increasing amount of γ-PGA or γ-PGA SAP content in the soil as can be seen from the Table 5. The yield of winter wheat increased by 7.62% (P40), 10.99% (P80), 13.14% (P120) and 14.67% (P160) compared to CK with the increasing of γ-PGA application in the soil, respectively. The yield of winter wheat increased by 4.85% (PS40), 7.15% (PS80), 8.74% (PS120) and 9.95% (PS160) compared to CK with the increasing of γ-PGA application in the soil, respectively. However, when the application amount of γ-PGA and γ-PGA SAP was more than 80 kg/hm^2^, the increasing rate of winter wheat yield decreased. In the treatments with the same amount of γ-PGA or γ-PGA SAP, the yields of winter wheat treated with γ-PGA in the soil were 0.75% (40 kg/hm^2^), 2.90% (80 kg/hm^2^), 2.43% (120 kg/hm^2^) and 2.55% (160 kg/hm^2^) higher than those treated with γ-PGA SAP in the soil, respectively.

#### Effect of γ-PGA and γ-PGA SAP for winter wheat quality

The effects of different contents of γ-PGA and γ-PGA SAP on the grain quality (protein, starch and reducing sugar) of winter wheat was shown in Table 6. The protein content of wheat grains increased somewhat with the increasing content of γ-PGA or γ-PGA SAP; however, there were no significant differences within each treatment. The protein content of wheat grains treated with γ-PGA was higher than that of wheat grains treated with γ-PGA SAP, which might be due to the fact that γ-PGA promotes the absorption and transformation of soil nitrogen by crops. However, there was no significant difference in starch content and reducing sugar content of wheat grains among the different treatments.

#### Effect of γ-PGA and γ-PGA SAP in the soil on soil water use efficiency (WUE) of winter wheat

The application of γ-PGA or γ-PGA SAP in the soil can improve WUE (Table 7), which was similar to the law of wheat yield. The addition of γ-PGA to soil increased WUE by 4.75% (P40), 8.07% (P80), 8.30% (P120), and 9.80% (P160) compared to the control group. And the WUE of addition γ-PGA SAP in the soil increased by 2.49% (PS40), 5.66% (PS80), 7.09% (PS120) and 8.37% (PS160) than the control group, respectively. However, the application of γ-PGA to soil increased WUE more than γ-PGA SAP. With γ-PGA SAP, the WUE in the soil was 2.16% (40 kg/hm^2^), 2.23% (80 kg/hm^2^), 1.11% (120 kg/hm^2^) and 1.30% (160 kg/hm^2^) lower than that treated with the same amount of γ-PGA in the soil, respectively.

**Table 7:** Water use efficiency and fertilizer partial productivity of each treatment.

Treatment	Yield (kg/hm^2^)	I(mm)	P(mm)	△W (mm)	WET (mm)	WUE(kg/(hm^2^ mm))
CK	5873.08±132.96b	270	60.5	112.31	442.81	13.26
P40	6182.73±384.05ab	270	60.5	114.68	445.18	13.89
P80	6327.35±298.69ab	270	60.5	111.06	441.56	14.33
P120	6422.74±483.95a	270	60.5	116.76	447.26	14.36
P160	6498.31±309.06a	270	60.5	115.93	446.43	14.56
PS40	6008.38±356.05ab	270	60.5	111.48	441.98	13.59
PS80	6177.61±481.37ab	270	60.5	110.51	441.01	14.01
PS120	6246.78±332.92ab	270	60.5	109.53	440.03	14.20
PS160	6305.49±263.81ab	270	60.5	108.28	438.78	14.37

## Discussion

It has been found that the average soil water content with the addition of γ-PGA in the soil was not significantly different from that of the control group or even decreased. However, the average soil water content increased significantly with the increase of γ-PGA SAP content in the soil [17]. The effects of γ-PGA and γ-PGA SAP on soil water content showed different changing trends, which was due to the γ-PGA could promote the growth of crop roots in the soil as a macromolecular fertilizer synergist and the developed crop roots could promote the absorption of soil water by crops [23]. Therefore, the soil water content in the γ-PGA treatment decreased compared with the control group. However, γ-PGA SAP is a three-dimensional polymer network structure that is insoluble in water, which can absorb water and swell in the soil to store water in the network structure, and it is not easily to lose water under a certain temperature and pressure [24]. Therefore, the soil water content of γ-PGA SAP treatment is higher than that of the control group.

The soil nitrate nitrogen and ammonium nitrogen contents significantly increased in the soil treated with γ-PGA compared to the control group. Moreover, the increase in the soil nitrate nitrogen and ammonium nitrogen content was proportional to the γ-PGA content in the soil [8]. It will be gradually decomposed into smaller molecular weight γ-PGA by the microorganisms in the soil over the time until to glutamic acid. The microorganisms then transform the glutamic acid into nitrate nitrogen and ammonium nitrogen [25]. The content of nitrate nitrogen in soil treated by γ-PGA SAP did not change significantly compared with the control group, while the content of ammonium nitrogen increased with the increasing of γ-PGA SAP content in the soil, but the increase of ammonium nitrogen content in the application of γ-PGA SAP in the soil was lower than that with the same amount of γ-PGA in the soil [4]. This is due to the γ-PGA SAP is a water-insoluble three-dimensional macromolecule network structure, although it will eventually be decomposed into small molecular weight γ-PGA or glutamic acid after its water retention failure [10]. Its degradation rate is significantly slower than that of γ-PGA single-chain polymer material soluble in water during the growth period of winter wheat. Therefore, γ-PGA SAP has little effect on soil nitrate nitrogen content. The γ-PGA SAP material has a negative charge after absorbing water and has a certain adsorption effect on ammonium nitrogen with positive charge. Consequently, the ammonium nitrogen content in the soil will increase proportionally with the γ-PGA SAP content in the soil.

The application of γ-PGA or γ-PGA SAP in the soil could significantly increase the number of soil microorganisms in each growth period. Furthermore, the number of microorganisms increased significantly with the amount of γ-PGA or γ-PGA SAP applied. The number of microorganisms with the same amount of γ-PGA in the soil was higher than that with the application of γ-PGA SAP in the soil. This is due to the fact that γ-PGA can be decomposed into glutamic acid in the soil and can be used as a nitrogen source by microorganisms. In the application of γ-PGA SAP in the soil, γ-PGA SAP could improve soil water conditions to provide a good breeding environment for soil microorganisms. Therefore, soil microorganisms also increased to a certain degree with the application of γ-PGA SAP in the soil. The increasing number of fungi with γ-PGA SAP addition in the soil was higher than that of actinomycetes and bacteria, which were due to the large individual volume of fungi, and the addition of γ-PGA SAP in the soil could increase the large pore volume of the soil [26]. Soil enzyme activity also showed an increasing trend with the application of γ-PGA and γ-PGA SAP in the soil, and the enzyme activity of the treatment with the same amount of γ-PGA was higher than that of the treatment with γ-PGA SAP in the soil. This was attributed to the formation of soil enzymes by microbial secretion or decomposition by their microorganisms. Therefore, enzyme activity was positively correlated with the number of soil microorganisms [27].

The yield of winter wheat increased with increasing γ-PGA and γ-PGA SAP applied to the soil under the same irrigation and fertilization conditions. This increase was primarily due to the fact that γ-PGA can increase the availability of nitrogen for crops in the soil. This, in turn, can increase fertilizer efficiency and promote nutrient absorption by crops, ultimately leading to an increase by adding γ-PGA to the soil [11, 23]. While the γ-PGA SAP can play a role in soil water and fertilizer conservation (Figs 3 and 4), which could promote the increase of crop yield. The yield of winter wheat treated with the same amount of γ-PGA was higher than that of the winter wheat treated with the same amount of γ-PGA SAP, which was due to the nutrient was the key factor to promote crop yield under normal irrigation level. The application of γ-PGA to the soil can increase the number of soil microorganisms and soil enzyme activity, promoting the conversion of γ-PGA to inorganic nitrogen. In addition, the absorption of amino acids by crops can enhance the stress resistance of crops, thus effectively promoting nutrient absorption and utilization by crops.

The application of γ-PGA in soil resulted in a certain degree of increase in grain protein. This increase can be attributed to the nitrogen source required for protein synthesis in grain, which includes nitrogen fertilizer and other types of organic matter in soil. Moreover, glutamic acid or other forms of nitrogen that are formed by the decomposition of urea and γ-PGA in the soil can also become a source of grain protein components.

## Conclusions

The application of γ-PGA and γ-PGA SAP can improve the soil microenvironment and increase the WUE of winter wheat, making it a promising approach for agricultural production.

The soil water content increased with an increasing amount of γ-PGA SAP applied to the soil. Additionally, the content of nitrate nitrogen and ammonium nitrogen increased with an increasing amount of γ-PGA applied to the soil. The content of ammonium nitrogen increased significantly with an increasing amount of γ-PGA SAP in the soil, while the γ-PGA SAP content in the soil did not significantly affect soil nitrate nitrogen. The number of soil microorganisms (bacteria, fungi and actinomycetes) and soil enzyme activities (urease, alkaline phosphatase and sucrase) also increased with the application of γ-PGA and γ-PGA SAP to the soil, with γ-PGA showing a higher abundance of both compared to γ-PGA SAP in the case of the same amount applied.

The yield of winter wheat increased with the application of γ-PGA and γ-PGA SAP in the soil, but the increasing rate of winter wheat yield decreased when the application amount of γ-PGA or γ-PGA SAP exceeded 80 kg/hm^2^, which increased by 5.95% and 6.77% compared with the control group, respectively. Interestingly, the addition of γ-PGA in the soil led to a significant increase in the protein content of wheat grains.
